# The Association of Serum Fibrinogen Levels With the Severity of Primary Postpartum Hemorrhage: A Prospective Study

**DOI:** 10.7759/cureus.71397

**Published:** 2024-10-13

**Authors:** Khushpreet Kaur, Seema Grover, Neetu Kukar, Isha Tapasvi, Meenali Garg, Richu Singla

**Affiliations:** 1 Department of Obstetrics and Gynecology, Guru Gobind Singh Medical College and Hospital, Faridkot, Faridkot, IND; 2 Department of Transfusion Medicine, Guru Gobind Singh Medical College and Hospital, Faridkot, Faridkot, IND; 3 Multidisciplinary Research Unit, Guru Gobind Singh Medical College and Hospital, Faridkot, Faridkot, IND

**Keywords:** association, coagulation disorder, fibrinogen, maternal mortality, obstetric emergency, postpartum hemorrhage

## Abstract

Introduction: Postpartum hemorrhage (PPH) remains a major cause of maternal mortality and morbidity worldwide. Coagulation disorders are a major risk factor for PPH that have not been studied adequately. Fibrinogen, a plasma glycoprotein, is integral to hemostasis and is involved in the final process of the coagulation cascade. Fibrinogen levels increase with advancing pregnancy probably because of an increase in estrogen levels and reach a peak (~5g/L) in the third trimester of pregnancy. However, in the postpartum period, the fibrinogen concentration shows a decreasing trend.

Objective: The purpose of the study was to study the serum fibrinogen level in patients with PPH and to find its association with the severity of primary postpartum hemorrhage.

Methodology: Patients were selected according to inclusion and exclusion criteria. Written and informed consents were taken. Routine investigations were done on admission and special investigations like red blood cell count, prothrombin time, international normalized ratio, activated partial thromboplastin time, and serum fibrinogen were done later when the patient developed PPH. Serial samples of all PPH patients for serum fibrinogen levels at 0, 1, 2, 4, and 24 hours were taken to study the serum fibrinogen levels and data were analyzed.

Result: The mean fibrinogen level on admission was 3.92 ± 0.92 g/l in non-severe PPH and 2.03 ± 0.97 g/l in the severe PPH group. Fibrinogen levels in the non-severe group remained < 4.46 g/l at 0, 1, 2, 4, and 24 hours. In the severe group, fibrinogen levels were < 2.89 g/l at 0, 1, 2, and 4 hours and it was 2.44 ± 1.06 g/l in the 24-hour samples. Statistical significance was found in all the fibrinogen values among the two study groups (p = 0.001).

Conclusion: Our results showed that zero-hour fibrinogen levels correlate significantly with the aggravation of PPH and levels <2 g/L can serve as a predictor for progression to severe PPH.

## Introduction

Postpartum hemorrhage (PPH) is an unpredictable obstetric emergency that is a direct cause of 1,32,000 maternal deaths worldwide every year [1]. Statistics reveal that of the total maternal deaths reported every year, 99% occur in developing countries and among these, 27.1% of deaths are due to PPH [2]. The incidence of PPH has decreased over the years but it continues to be a nightmare for obstetricians and adds significant morbidity to the patient and emotional, physical, and socio-economic burden to family.

The World Health Organization (WHO) defines PPH as “blood loss greater than or equal to 500 ml within 24 hours after birth”, irrespective of the mode of delivery, and severe PPH as “blood loss greater than or equal to 1,000 ml within 24 hours of childbirth” [2]. Primary (early) PPH occurs within the first 24 hours of delivery and is the most common cause of morbidity and mortality. Secondary (late) PPH occurs after 24 hours up to 12 weeks of delivery.

“The 4Ts” introduced by the American Academy of Family Physicians in the American Life Support in Obstetrics (ALSO) represent the summary of causes of PPH: tone (uterine atony), trauma of the birth canal, tissue retention from the placenta/fetus, and thrombin (bleeding disorders). Even though the etiology and risk factors have been discussed in the literature in depth, about two-thirds of the women who experience PPH have no identifiable clinical risk factors [3]; hence, it becomes difficult to predict PPH.

Pregnancy is a state of balanced hypercoagulability and is characterized by physiological anemia, neutrophilia, mild thrombocytopenia, increased procoagulant factors, and diminished fibrinolysis. These changes are due to an increase in estrogen levels with increasing gestational age. Pregnancy-related coagulation changes are expressed by a progressive and significant increase in the fibrinogen level, while the standard indicators such as prothrombin time (PT) and activated coagulation time (ACT), do not undergo much change [4]. Disorders of coagulation, even though a major risk factor for PPH, have not been studied adequately. In relation to PPH, coagulopathy falls into three major categories: inherited deficiencies, pregnancy-related hemorrhage, and acquired coagulopathy-like liver disorders [5]. The etiology of a severe case of PPH is mostly multifactorial and acquired deficiencies are becoming somewhat more frequent.

Fibrinogen, a plasma glycoprotein, is integral to hemostasis and is involved in the final process of the coagulation cascade. Fibrinogen (coagulation factor I) is present in the blood with a normal non-pregnant level of 2.0-4.5 g/L [6]. Fibrinogen levels increase with advancing pregnancy probably because of an increase in estrogen levels and reach a peak (~5g/L) in the third trimester of pregnancy [2,7]. In the postpartum period, the fibrinogen concentration shows a decrease and may remain low during the immediate puerperium [8]. During bleeding, fibrinogen causes platelet activation and aggregation by binding to glycoprotein IIb/IIIa receptors on platelet surfaces (primary hemostasis) and initiates fibrin polymerization after undergoing cleavage by thrombin (secondary hemostasis) [9]. During the first few hours after delivery, a marked increase in clotting system activity as well as fibrinolytic activity has been reported [10]. Disseminated intravascular coagulation (DIC) is a well-known phenomenon in the course of PPH. Intravascular fibrin deposition increases thereby increasing fibrinogen consumption [11] and hence low fibrinogen levels. The resultant bleeding is, however, quickly taken care of by the tonic uterus of a physiological pregnancy. In PPH, the hormonal balance regulating the terminal pregnancy sequence seems to be altered. Decreased fibrinogen levels may lead to its early initiation [12]. It has been proposed that the pathological increase in fibrinogen lysis and fibrinolysis may decrease fibrinogen levels and contribute to PPH. Recent studies have suggested that the limiting concentration of fibrinogen to maintain adequate hemostasis is 2 g/L [13]. When fibrinogen values drop to less than 1 g/L, it is associated with the loss of 1.4 blood volumes, establishing a relationship between fibrinogen values and the severity of PPH. Fibrinogen concentrate is widely used to correct acquired hypofibrinogenemia, but evidence is lacking regarding the efficacy of this treatment [14].

The evaluation of postpartum bleeding includes clinical, hemodynamic, and laboratory variables which are used to classify the severity of PPH. These parameters also help to assess patients at risk of hemorrhagic shock who may benefit from early blood transfusions and admission to the high-dependency unit (HDU) for close monitoring [15].

Active management of the third stage of labor (AMTSL) is the core prevention step. AMTSL is a feasible, low-cost measure to prevent 60-70% of atonic PPH [16]. In case of persistent atony, PPH is managed as per WHO 2020 guidelines, i.e. giving uterotonics, mechanical interventions, and surgical methods in case of failure of medical management [16]. In refractory PPH, the use of recombinant activated factor VII, desmopressin acetate, and fibrinogen concentrates has been recommended recently.

At present, there is limited research on hemostasis-related factors in PPH. Charbit et al. published one of the earliest studies profiling changes in fibrinogen levels during the course of PPH and probed serum fibrinogen to be the sole laboratory parameter independently associated with severe PPH [11]. Since then, many studies have supported the same while some have refuted the evidence. 

## Materials and methods

This was a prospective study carried out over a period of 18 months, from May 1, 2021, to October 31, 2022, at Guru Gobind Singh Medical College and Hospital, Faridkot, Punjab, India. The study was approved by the Thesis and Ethical Committee, Guru Gobind Singh Medical College and Hospital (approval number: BFUHS/2K22p-TH/7400). The study population included women who developed primary PPH after vaginal delivery. Exclusion criteria included women with gestation < 24 weeks, who had PPH after 24 hours, who received blood transfusion 24 hours before delivery, and those who did not give consent. A non-random convenient sampling technique was adopted, and a total of 40 eligible participants were considered for the study.

PPH management

AMTSL was done in all patients to prevent PPH. If excessive bleeding was observed, uterine massage was done and uterotonics were given. Injection carboprost 0.25mg i.m. was given (every 15-20 minutes where required). Injection methergine 0.2 mg i.m. or tab misoprostol 800 μg was given, wherever needed.

In cases where medical management was unsuccessful, mechanical interventions like uterine balloon tamponade (UBT), bimanual uterine compression, hemostatic intrauterine packing, or non-pneumatic anti-shock garments (NASG) were used, and if they failed, surgical interventions like arterial ligation, obstetric hysterectomy were undertaken. Fluid resuscitation and blood products were given as required.

Methodology

The patients were explained about the procedure and written consent was taken from them. According to the severity of bleeding over the course of 24 hours, the patients were divided into two groups: severe PPH and non-severe PPH. Severe PPH was defined by the occurrence of one of these events: (i) Peripartum hemoglobin decrease > 4 g/l, (ii) Transfusion of packed red blood cells, (iii) Arterial embolization or emergency surgeries like hysterectomy, arterial ligation, and (iv) Admission to intensive care or death. Patients with blood loss < 500 ml and who did not fulfill the criteria of severe PPH were classified under non-severe PPH [8].

Blood loss was estimated by using the trays provided under our delivery tables. The wet mops were squeezed into the tray. The blood collected in a tray was put into a calibrated jar and measured. 

Data pertaining to demographics, medical history, past obstetric history, and routine investigations on admission were collected using self-structured proforma which consisted of detailed history, investigation, and treatment modalities used.

An additional blood sample of 2 ml was preserved in an ethylenediamine tetraacetic acid (EDTA) vial in the hematology lab for additional biomarkers which were processed if the patient had PPH. These investigations included red blood cell count, prothrombin time expressed as international normalized ratio (INR), serum fibrinogen, and activated partial thromboplastin time (aPTT).

At the onset of PPH after uterine massage and carboprost administration, the first sample for serum fibrinogen level (described as zero-hour sample (H0)) was taken followed by serial samples at 1, 2, 4, and 24 hours. Serum fibrinogen levels were measured by the manual tyrosine method. The data was analyzed, and serum fibrinogen cut-off values were defined for severe and non-severe PPH groups. These fibrinogen levels may serve as an alarm for the obstetrician for severe PPH.

Procedure for Fibrinogen Estimation (Manual Tyrosine Method)

The blood samples (2 ml each) were collected in EDTA vials and centrifuged at 2000 rpm for 15 minutes for separation of plasma. a 0.5 ml of plasma was added to the plasma, followed by the addition of 14 ml distilled water and 0.5 ml of calcium chloride. The solution was incubated at 37^ o^C till clot formation. The clot was washed thoroughly with water and added to 5 ml of heated sodium hydroxide (NaOH). It was further boiled till the clot dissolved. The pH of the solution was checked with a litmus paper and neutralized with sulfuric acid (H_2_SO_4_) to get a final pH of 7. To this, 0.5 ml of Folin Ciocalteau reagent and sodium carbonate (Na_2_CO_3_) (3ml) were added. The solution was made up to 25 ml with distilled water and placed in an incubator at 37 ^o^C for one hour followed by reading at 680 nm with water as blank. The obtained reading was entered into the given formula and the concentration of fibrinogen was obtained in g/L.

Plasma fibrinogen = (Reading of unknown x 200/ Reading of standard x 1000) x 16.4

 = (Reading of unknown x Reading of standard) x 3.28

Statistical analysis

The collected data were compiled and entered into Microsoft Excel (Microsoft Corporation, Redmond, Washington, United States). Statistical analyses were performed using IBM SPSS Statistics for Windows, Version 26.0 (Released 2019; IBM Corp., Armonk, New York, United States).

## Results

We included 40 patients in our study. PPH was severe in 17 patients and non-severe in 23 patients. Nearly two-thirds of the patients in both groups were of term gestation. In the non-severe group, 12 (52.2%) patients were nulliparous, and one (4.3%) was grand multipara whereas in the severe PPH group, only four patients (23.5%) were nulliparous and the remaining patients (76.5%) were multiparous.

Risk factors were identified in 56.5% (n=13) of patients in the non-severe group and in 58.8% (n=13) of patients in the severe PPH group (Table 1). However, no statistical significance was found among the two groups (p = 0.884). Anaemia was identified as the most common risk factor in the non-severe group (56.5%) followed by multiparity in 47.8% of patients. The most common risk factor in the severe PPH group was multiparity (76.5%) followed by anemia found in 47.1% of patients. No statistical significance was found between the two study groups.

**Table 1:** Association of risk factors with severity of PPH HDP: hypertensive disorders of pregnancy; IHCP: intrahepatic cholestasis of pregnancy; LGA: large for gestational age; IUD: intrauterine demise; PPROM: preterm pre-labor rupture of membranes; GDM: gestational diabetes mellitus; PPH: postpartum hemorrhage

Risk Factor	Non-Severe PPH (n = 23)		Severe PPH (n = 17)		Total, n	Chi Square Value	p-value
	Frequency	Percentage	Frequency	Percenatage	Frequency		
None	10	43.40%	04	23.50%	14	1.563	0.325
Multiparity	11	47.80%	13	76.50%	24	3.342	0.104
Anemia	13	56.50%	08	47.10%	21	0.351	0.554
Thrombocytopenia	07	30.40%	05	29.40%	12	4.224	0.61
Prolonged first stage of labor	04	17.40%	05	29.40%	09	0.810	0.368
HDP	05	21.70%	00	0.00%	05	0.775	0.379
IHCP	00	0.00%	03	17.60%	03	4.388	0.036
Jaundice	02	8.70%	01	5.90%	03	0.112	0.738
LGA baby	02	8.70%	00	0.00%	02	1.556	0.212
IUD (macerated)	01	4.30%	02	11.80%	03	1.556	0.212
PPROM	01	4.30%	02	11.80%	03	0.775	0.379
Teenage pregnancy	02	8.70%	00	0.00%	02	2.397	0.122
Multiple gestation	01	4.30%	01	5.90%	02	0.048	0.826
GDM	01	4.30%	00	0.00%	01	0.758	0.384
Elderly primigravida	01	4.30%	00	0.00%	01	0.758	0.384
Fibroid uterus	00	0.00%	01	5.90%	01	1.388	0.239

Labor onset was spontaneous in most of the cases in both groups. Induction was required in 17.30% (n = 4) of cases in the non-severe and 17.60% (n=3) of cases in the severe PPH group. In the non-severe group, 12 patients (52.2%) required induction/augmentation of labor as compared to 11 patients (64.7%) in the severe PPH group. Misoprostol was the most common drug used for induction in both the groups whereas one patient in the non-severe group was induced with prostaglandin E2 gel. No statistical significance was found between the two groups (p = 0.658).

The mean duration of the first stage of labour was 12.88 ± 6.94 hours in the non-severe group and 12.65 ± 7.46 hours in the severe group without any statistical significance (p = 0.812). The mean duration of second-stage labor was 1.42 ± 0.80 hours in the non-severe group and 0.94 ± 0.61 hours in the severe group with statistical significance (p = 0.045). The mean duration of the third stage of labor was shorter in the non-severe group (13.35 ± 4.05 min) as compared to the severe group (14.65 ± 5.17 minutes) with no statistical significance in the two groups (p = 0.378). The mean duration of total labor was 14.30 ± 7.44 hours in the non-severe group and 13.71 ± 8.28 hours in the severe group. The analysis found no statistical significance between the two groups (p=0.812).

The majority of patients had a normal vaginal delivery (NVD) in both groups (Table 2). Preterm delivery occurred in seven patients and three patients had outlet forceps delivery. However, there was no statistical significance with respect to the mode of delivery between the two groups (p = 0.507).

**Table 2:** Association of mode of delivery with severity of PPH PPH: postpartum hemorrhage

Mode of Delivery	Non-Severe PPH (n = 23)		Severe PPH (n = 17)		Total	Chi-Square Value	p-value
	Frequency	Percentage	Frequency	Percentage	Frequency		
Normal vaginal delivery	08	34.80%	10	58.80%	18	2.327	0.507
Normal vaginal delivery + Right mediolateral episiotomy	08	34.80%	04	23.50%	12		
Preterm vaginal delivery	05	21.70%	02	11.80%	07		
Forceps + Right mediolateral episiotomy	02	08.70%	01	05.90%	03		

There was no trauma during delivery in the majority of cases in both groups. Cervical tears were present in six patients (13.0% (n=3) in the non-severe group and 17.6% (n=3) in the severe PPH group). Episiotomy was given in 43.5% (n=10) of patients in the non-severe and 23.4% (n=5) in the severe PPH group. Vaginal laceration was found in only one patient in the non-severe group. There was no statistical difference among the two groups (p = 0.645).

All the patients in the non-severe group had blood loss < 1000 ml whereas in the severe group, only one patient belonged to this category (Table 3). Mean blood loss in the non-severe group was 752.17 ± 77.52 ml and it was 1320.59 ± 291.58 ml in the severe group. Statistically, this difference was highly significant (p = 0.001).

**Table 3:** Association of blood loss with severity of PPH PPH: postpartum hemorrhage

Blood Loss (ml)	Non-Severe PPH (n = 23)		Severe PPH (n = 17)		Total	Chi-Square Value	p-value
	Frequency	Percentage	Frequency	Percentage	Frequency		
500-1000	23	100.00%	01	5.90%	24	36.708	0.001
1000-1500	00	0.00%	12	70.60%	12		
1500-2000	00	0.00%	04	23.50%	04		

All the patients in the severe PPH group had blood transfusions as compared to only 13.0% (n=3) of patients in the non-severe group. Analysis revealed a statistical significance in two groups (p = 0.001). Out of the total 40 patients, 20 received blood products. None of the patients in the non-severe group received PRBC transfusion as compared to 100% in the severe group. Fresh frozen plasma was given to 16 and platelet concentrates to six patients (including single donor platelet (SDP) to one patient). The study revealed a statistical significance in the two groups (p = 0.001).

AMTSL was done in all patients. During medical management, tab misoprostol was the most common drug used in both groups (91.3% (n=21) in the non-severe group and 100% (n=17) in the severe group). In the non-severe group, all cases were successfully managed with medical management alone (Table 4). In the severe group, the UBT and NASG (29.4%) were the most commonly used mechanical methods after the failure of medical therapy. Despite the above measures, one patient in the severe PPH group had to undergo obstetric hysterectomy.

**Table 4:** Treatment modalities used in PPH PPH: postpartum hemorrhage

Treatment Modality Used	Non-Severe PPH (n = 23)		Severe PPH (n = 17)		Total	Chi-Square Value	p-value
	Frequency	Percentage	Frequency	Percentage	Frequency		
Tab Misoprostol	21	91.30%	17	100.0%	38	1.556	0.499
Inj. Carbeprost	18	78.30%	16	94.10%	34	1.928	0.216
Inj. Oxytocin	18	78.30%	13	76.50%	31	0.018	0.893
Inj. Methergin	10	43.50%	12	70.60%	22	2.903	0.116
Surgical suturing of lesions	14	60.90%	08	47.10%	22	0.753	0.385
Inj. Tranexamic acid	04	17.40%	12	70.60%	16	11.526	0.001
Inj. Carbetocin	05	21.70%	04	23.50%	09	0.018	0.893
Uterine balloon tamponade	00	0.00%	05	29.40%	05	7.731	0.009
Non-pneumatic anti-shock garment	00	0.00%	05	29.40%	05	7.731	0.009
Intrauterine packing	00	0.00%	04	23.50%	04	6.013	0.026
Intravaginal packing	00	0.00%	03	17.60%	03	4.388	0.069
Obstetric hysterectomy	00	0.00%	01	5.90%	01	1.388	0.425

All the patients in the non-severe PPH were effectively managed in the ward whereas eight patients in the severe PPH group (47.1%) required admission to the ICU. A statistical difference was found among the two groups (p = 0.001). All the patients in the non-severe group were discharged without any complications as compared to only three patients (17.6%) in the severe PPH group. A total of 12 patients (70.6%) were near-miss mortality and two patients (11.8%) had mortality in the severe PPH group. A statistical difference was found among the two study groups (p = 0.001).

The mean hemoglobin values at zero, four, and 24 hours were 10.41 ± 1.11, 9.03 ± 0.72, and 9.09 ± 0.87 g/dL, respectively, in the non-severe group and 10.28 ± 1.99, 7.46 ± 1.17, and 8.70 ± 0.93 g/dL, respectively, in the severe group. The mean total leukocyte count (TLC) values at zero, four, and 24 hours were 11.89 ± 4.0, 16.31 ± 3.94, and 11.44 ± 1.86 x 103/mm^3^, respectively, in the non-severe group and 13.08 ± 5.80, 17.69 ± 4.12, and 13.67 ± 3.66 x 103/mm^3^, respectively, in the severe group. The mean platelet counts at zero, four, and 24 hours were 1.75 ± 0.84, 1.70 ± 0.74, and 1.82 ± 0.83 lac/mm^3^, respectively, in the non-severe group and 1.52 ± 0.73, 1.62 ± 0.58, and 1.61 ± 0.58 lac/mm^3^, respectively, in the severe group. The mean value of four-hour hemoglobin and mean 24-hour TLC values were of statistical significance (p = 0.001 and 0.016, respectively). Among the special blood investigations, in the non-severe and severe groups, the mean RBC count was 3.66 ± 0.44 and 3.53 ± 0.63 million/mm^3^, respectively; the mean BT was 13.0 ± 0.80 and 12.53 ± 0.80 seconds, respectively; the mean PT was 87.45 ± 3.98 and 88.56 ± 5.01 seconds, respectively; the mean PTi-INR was 1.01 ± 0.06 and 0.98 ± 0.06, respectively; and the mean aPTT was 29.87 ± 2.14 and 35.94 ± 3.51 seconds, respectively. None of the special investigations except aPTT showed any statistical significance (p = 0.001).

**Figure 1:**
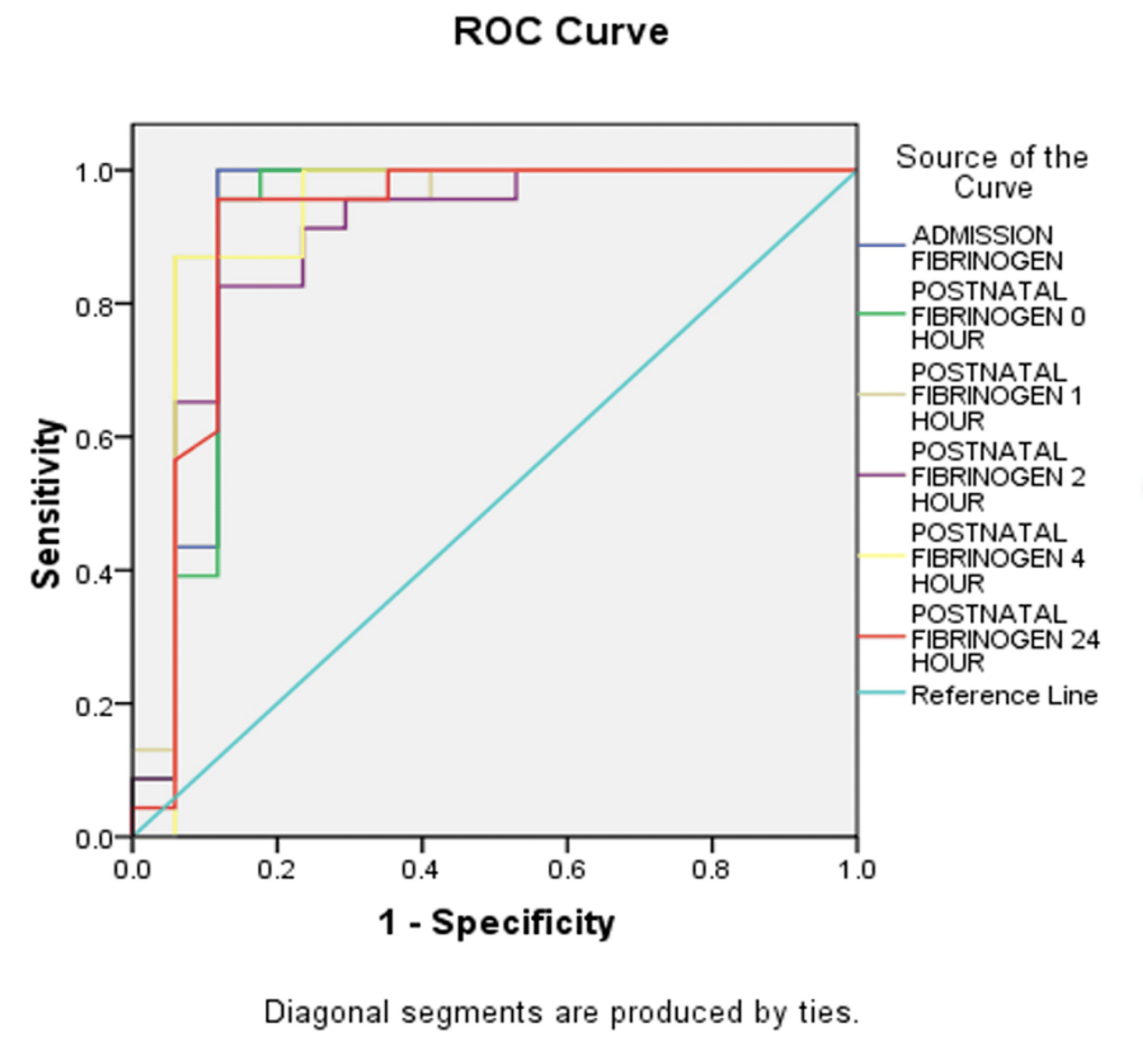
AUC curve for fibrinogen levels at different hours of PPH ROC: receiver-operating characteristic; AUC: area under the curve; PPH: postpartum hemorrhage

During the measurements, the fibrinogen level was found to be 2.35 g/L at zero hour with 96% sensitivity and 88.2% specificity, 2 g/L at one hour with 96% sensitivity and 88.2% specificity, 2.1 g/L at two hours with 91.3% sensitivity and 77% specificity, 2.87 g/L at four hours with 87% sensitivity and 94% specificity, and 2.97 g/L at 24 hours with 95.7% sensitivity and 88.2% specificity (Figure 1). 

**Figure 2:**
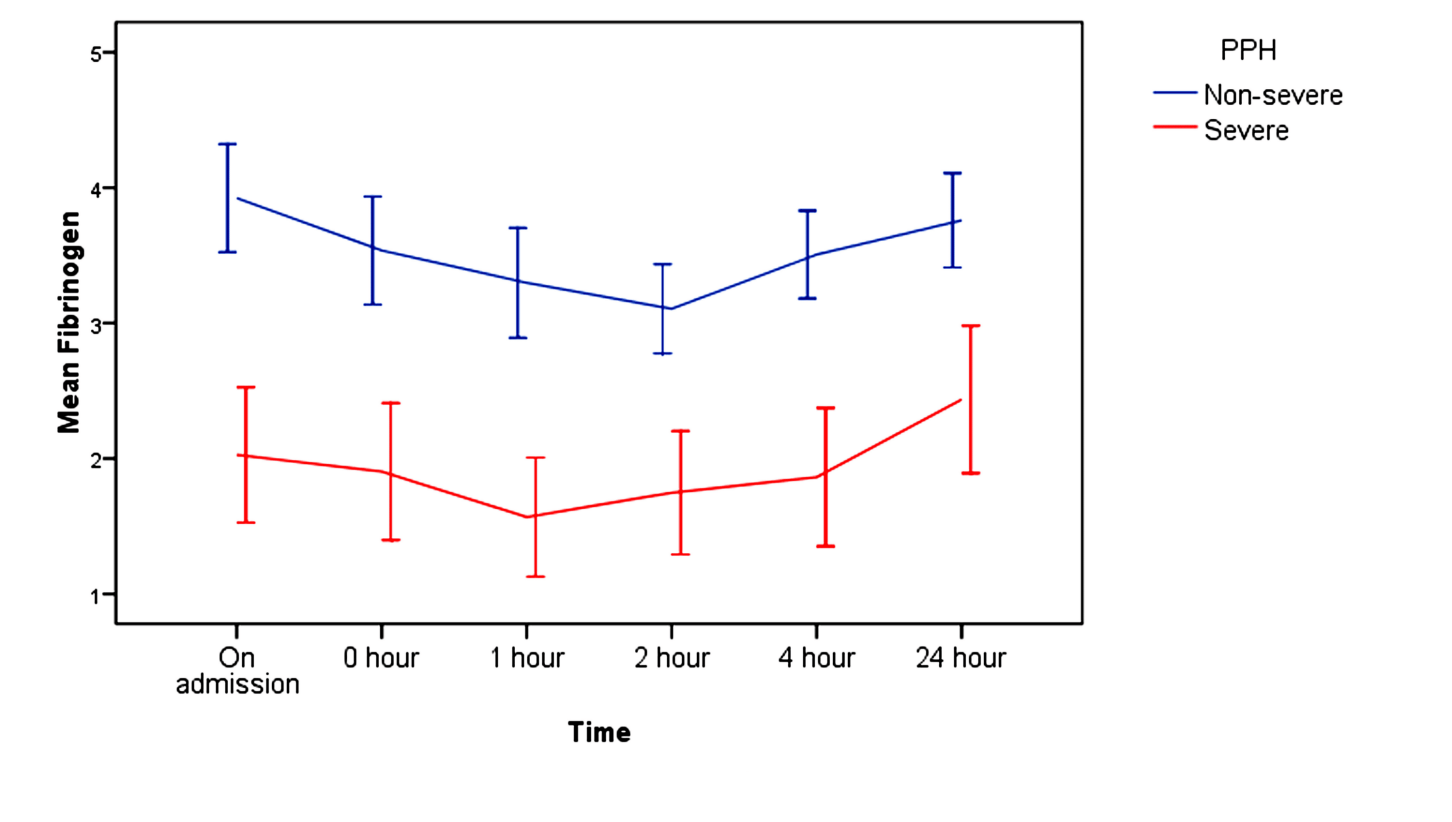
Association of fibrinogen levels with time PPH: postpartum hemorrhage

The mean fibrinogen values at zero, one, two, four, and 24 hours were 3.54 ± 0.92, 3.30 ± 0.94, 3.11 ± 0.76, 3.51 ± 0.75, 3.76 ± 0.80g/L, respectively, in the non-severe group and 1.90 ± 0.99, 1.57 ± 0.86, 1.75 ± 0.89, 1.86 ± 0.99, 2.44 ± 1.06 g/L, respectively, in the severe group (Table 5). Fibrinogen levels in the non-severe group remained < 4.46 g/l at zero, one, two, four, and 24 hours. In the severe group, fibrinogen levels were < 2.89 g/l at zero, one, two, and four hours and it was 2.44 ± 1.06 in the 24-hour samples (Figure 2). Statistical significance was found in all the fibrinogen values among the two study groups (p = 0.001).

**Table 5:** Association of serum fibrinogen levels with severity of PPH PPH: postpartum hemorrhage

Fibrinogen Levels	Non-Severe PPH		Severe PPH		Z value	p-value
	Mean	SD	Mean	SD		
On Admission	3.92	0.92	2.03	0.97	6.285	0.001
Postnatal 0 hour	3.54	0.92	1.90	0.99	5.374	0.001
Postnatal 1 hour	3.30	0.94	1.57	0.86	5.979	0.001
Postnatal 2 hour	3.11	0.76	1.75	0.89	5.197	0.001
Postnatal 4 hour	3.51	0.75	1.86	0.99	5.967	0.001
Postnatal 24 hour	3.76	0.80	2.44	1.06	4.494	0.001

## Discussion

PPH being a significant cause of maternal morbidity and mortality worldwide, necessitates a deeper understanding of its risk factors and underlying mechanisms. Fibrinogen, a crucial protein in the coagulation cascade, is essential for clot formation and stability. Recent studies suggest that both low and high fibrinogen levels may be linked to varying degrees of PPH, influencing both the risk of bleeding and the effectiveness of hemostatic interventions. Understanding how varying fibrinogen concentrations may correlate with PPH severity could provide valuable insights into risk stratification and management strategies. This discussion aims to delve into existing research to elucidate the potential of serum fibrinogen as a predictive marker in assessing the severity of PPH and its implications for maternal care. 

The mean fibrinogen level during the ninth month of pregnancy is 5 g/L [7]. During PPH, the fibrinogen level decreases rapidly, influenced by two principal mechanisms: the blood loss itself, which induces depletion of coagulation factors, and the consumption of factors associated with coagulation activation [8]. In our study, the mean fibrinogen level on admission was 3.92 ± 0.92 g/l in the non-severe and 2.03 ± 0.97 g/l in the severe PPH group. The difference among the two groups was statistically significant. Kender et al. found that fibrinogen level was a predictor of blood loss ≥ 500 mL (OR = 0.996, 95%CI = 0.993-0.999, P = 0.01). To detect the PPH ≥ 500 mL after vaginal delivery, they calculated the threshold of prepartum fibrinogen level as 452 mg/dL with a sensitivity of 66.1% and a specificity of 57.1% [17]. Another study done by Agarwal et al. found mean fibrinogen levels of 2.80 ± 5.49 g/L in the non-severe group and 1.07 ± 4.78 g/L in the severe group with p < 0.001, which was statistically highly significant [12]. In a study by Wang et al. in Japan in 2016, lower fibrinogen levels were found to be an independent risk factor for PPH and showed a negative correlation with estimated blood loss [18]. Charbit et al. also found that the mean fibrinogen value in the non-severe group was 4.4 ± 3.7 and in the severe group, it was 3.3 ± 2.5 with p < 0.001 [11]. Kaufner et al. studied 217 women who underwent vaginal delivery and found no correlation between serum fibrinogen levels and postpartum blood loss [19]. Women with and without PPH did not differ much in mean serum fibrinogen levels [19]. Karlsson et al. also could not find an association between the prepartum fibrinogen levels and PPH. They defined PPH as blood loss > 1000 mL and they included both cesarean and vaginal deliveries in their study, unlike our study [20]. Yamada et al. were unable to report a significant correlation between antenatal fibrinogen concentrations and estimated blood loss in their retrospective study of vaginal deliveries. It included women without PPH risk factors, but they checked the fibrinogen level 21 days before delivery [21].

In our study, fibrinogen levels were measured at zero, one, two, four, and 24 hours from the onset of PPH. During the measurements, the fibrinogen level was found to be 2.35 g/L at zero hours with 96% sensitivity and 88.2% specificity, 2 g/L at one hour with 96% sensitivity and 88.2% specificity, 2.1 g/L at two hours with 91.3% sensitivity and 77% specificity, 2.87 g/L at four hours with 87% sensitivity and 94% specificity, and 2.97 g/L at 24 hours with 95.7% sensitivity and 88.2% specificity. The mean fibrinogen values at zero, one, two, four, and 24 hours were 3.54 ± 0.92, 3.30 ± 0.94, 3.11 ± 0.76, 3.51 ± 0.75, 3.76 ± 0.80 g/L, respectively, in the non-severe group and 1.90 ± 0.99, 1.57 ± 0.86, 1.75 ± 0.89, 1.86 ± 0.99, 2.44 ± 1.06 g/L, respectively, in the non-severe group. This difference in the mean fibrinogen values among the two groups was statistically significant (p = 0.001). Charbit et al. observed that mean fibrinogen values at H0, H1, H2, and H4 were 2.63 ± 1.66, 2.70 ± 1.75, 3.70 ± 2.14, 5.00 ± 2.633 g/L, respectively. The p-values were <0.001 which were highly statistically significant [11]. Cortet et al. measured fibrinogen levels at the same intervals as in our study [22]. They concluded that the zero-hour fibrinogen is a marker of the risk of aggravation of PPH and should serve as an alert to clinicians. This study found that there is a higher risk of severe PPH when fibrinogen is <2 g/L. In the study by Zakaria et al., the mean serum fibrinogen levels at zero hour in group A was 4.2 ± 1.2 SD and in group B it was 3.4 ± 0.9 SD with a p-value equal to 0.002 [23]. Comparison between both groups showed a highly significant difference. They postulated that during severe PPH, fibrinogen is the factor that decreases fastest and thus, leads to further reduction in fibrinogen levels. The only coagulation variable that remained independently associated with severe hemorrhage was the fibrinogen level. In their study, a fibrinogen level below 2 g/L multiplied the risk of severe PPH by 11 times. In our study, all patients with serum fibrinogen levels <2 g/L at zero hour developed severe PPH. Thus, fibrinogen levels <2 g/L can serve as a predictor for severe hemorrhage. This association between reduced levels of zero-hour fibrinogen and the severity of PPH was also found by Cortet et al. [22] and Zakaria et al. [23]. However, all patients with severe PPH did not have their fibrinogen levels <2 g/L. Thus, it indicates that severe PPH can occur even with a mild reduction in fibrinogen levels (2-3 g/L). No cut-off could be defined for fibrinogen levels to predict non-severe PPH. Cortet et al. also found that fibrinogen levels between 2 and 3 g/L are associated with a nearly doubled risk of severe hemorrhage and may serve as a warning sign. [22]

In our study, risk factors were present in more than half of the patients. Multiparity and anemia were the two most common risk factors. These findings were corresponding to a prospective study on primary PPH in a tertiary care center in Telangana, India [24]. Other risk factors in our study were thrombocytopenia, prolonged first stage of labor, induction/augmentation of labor, intrahepatic cholestasis of pregnancy (IHCP), jaundice, PPROM, intrauterine demise (IUD) baby, teenage pregnancy, multiple gestation, large for gestational age (LGA) baby, gestational diabetes mellitus (GDM), elderly primigravida, and myoma uterus. In the present study, there were 52.17% (n=12) nulliparous and 47.82% (n=11) multiparous patients in the non-severe group whereas in the severe group, there were 23.5% nulliparous and 76.5% multiparous patients. A study done on PPH in Japan in 2016 found that PPH was more common in nulliparous than multiparous women (16%, n=77/474 vs. 11%, n=50/475) and it was an independent risk factor for PPH [25]. However, these findings were in contrast to those obtained by other authors [11,23,22,26,27]. 

In the present study, mean blood loss in the non-severe group was 752.17 ± 77.52 ml as compared to 1320.59 ± 291.58 ml in the severe group. All patients in the severe PPH group had blood transfusions as compared to only 13.0% of patients in the non-severe group. These findings were close to those observed in a study by Velasquez et al. (88.2% of patients in the severe group and 12.9% of patients in the non-severe group) [28]. In the non-severe group, all cases were successfully managed with medical management alone. In the severe PPH group, patients not responsive to medical therapy were managed with mechanical methods like UBT, NASG, and packing. Despite the above measures, one patient required obstetric hysterectomy. All the patients with non-severe PPH were effectively managed in the ward whereas eight patients in the severe PPH group (47.1%) required admission to the ICU. The mean duration of ICU stay was 31.5 hours which was close to that observed in a study in Spain [27].

In our study, 12 patients (70.6%) were near-miss mortality and there were two maternal mortalities in the severe PPH group. The latter two patients had multiple obstetric and medical risk factors. The first patient was a multipara with preterm twin gestation and came in active labor. She was moderately anemic, had thrombocytopenia and prolonged aPTT, and was managed by a multi-disciplinary team. She was given uterotonics followed by UBT and NASG. A total of five units of PRBC, five units of PC, and eight units of FFP were given. She had an ICU stay of 46 hours before death occurred due to irreversible shock. The second patient was a term multiparous patient with IHCP and developed atonic PPH which couldn't be managed with conservative methods and an obstetric hysterectomy was done. She was given three units of PRBC, six of PC, eight of FFP, and an ICU stay of 14 hours. She died due to sequelae of sepsis and acute respiratory distress syndrome (ARDS). It was noted that the fibrinogen level of both these patients was <2 g/L at zero hours of PPH. 

In the current study, the mean aPTT was 29.87 ± 2.14 in the non-severe and 35.94 ± 3.51 in the severe group (p = 0.001). The mean values of aPTT were statistically significant in our study as well as the study by Cortet et al. [22], whereas mean hemoglobin and platelet values were not significant. A study done on hemostatic tests following a major hemorrhage in 2011 in the United Kingdom showed no correlation between aPTT and blood loss [29]. In the present study, the mean PT was 87.45 ± 3.98 in the non-severe and 88.56 ± 5.01 in the severe group (p = 0.483) and the mean INR was 1.11 ± 0.06 in the non-severe and 1.08 ± 0.06 in the severe group (p = 0.135). Our parameters were comparable with a study by Charbit et al. [11] and were not significant statistically. However, owing to limited resources and being a tertiary center in a remote area, only a few coagulation parameters could be studied. Factor VII, vWF, and other concomitant parameters could not be evaluated. This is a limitation of our study. Also, the sample size was relatively small. Hence, we suggest a multi-centric study for the evaluation of fibrinogen levels along with other concomitant coagulation factors. The strengths of our study were a matched cohort of pregnant women with similar induction methods in both groups, and AMTSL, and serial measurement of postpartum fibrinogen levels. This study was undertaken to find out if there was an association between serum fibrinogen levels and the severity of PPH following vaginal delivery. Our study can serve as a guide to identify patients who are likely to have severe PPH so that blood products can be arranged and we can be prepared for all medical and surgical measures without losing time.

## Conclusions

The need to find a parameter for PPH has always existed as most of the PPH cases occur without any warning and may result in bad outcomes. According to our study, zero-hour fibrinogen levels correlate significantly with the aggravation of primary PPH and levels <2 g/L can serve as a predictor for progression to severe PPH. This parameter will be helpful, especially in unpredictable cases of PPH, for better preparedness towards transfusions and definitive management. Hence, early recognition of this coagulopathy is crucial for successful PPH management.
